# The intron binding protein EMB-4 is an opposite regulator of cold and high temperature tolerance in *Caenorhabditis elegans*

**DOI:** 10.1093/pnasnexus/pgae293

**Published:** 2024-07-26

**Authors:** Akane Ohta, Yuki Sato, Kazuho Isono, Takuma Kajino, Keisuke Tanaka, Teruaki Taji, Atsushi Kuhara

**Affiliations:** Graduate School of Natural Science, Konan University, 8-9-1 Okamoto, Higashinada-ku, Kobe, Hyogo 658-8501, Japan; Faculty of Science and Engineering, Konan University, 8-9-1 Okamoto, Higashinada-ku, Kobe, Hyogo 658-8501, Japan; Institute for Integrative Neurobiology, Konan University, 8-9-1 Okamoto, Higashinada-ku, Kobe, Hyogo 658-8501, Japan; Graduate School of Natural Science, Konan University, 8-9-1 Okamoto, Higashinada-ku, Kobe, Hyogo 658-8501, Japan; Institute for Integrative Neurobiology, Konan University, 8-9-1 Okamoto, Higashinada-ku, Kobe, Hyogo 658-8501, Japan; Department of Bioscience, Tokyo University of Agriculture, 1-1-1 Sakuragaoka, Setagaya-ku, Tokyo 156-8502, Japan; Department of Bioscience, Tokyo University of Agriculture, 1-1-1 Sakuragaoka, Setagaya-ku, Tokyo 156-8502, Japan; NODAI Genome Research Center, Tokyo University of Agriculture, 1-1-1 Sakuragaoka, Setagaya-ku, Tokyo 156-8502, Japan; Department of Bioscience, Tokyo University of Agriculture, 1-1-1 Sakuragaoka, Setagaya-ku, Tokyo 156-8502, Japan; Graduate School of Natural Science, Konan University, 8-9-1 Okamoto, Higashinada-ku, Kobe, Hyogo 658-8501, Japan; Faculty of Science and Engineering, Konan University, 8-9-1 Okamoto, Higashinada-ku, Kobe, Hyogo 658-8501, Japan; Institute for Integrative Neurobiology, Konan University, 8-9-1 Okamoto, Higashinada-ku, Kobe, Hyogo 658-8501, Japan; AMED-PRIME, Japan Agency for Medical Research and Development, 1-7-1 Otemachi, Chiyoda-ku, Tokyo 100-0004, Japan

**Keywords:** *Caenorhabditis elegans*, cold tolerance, heat tolerance, RNA-binding protein

## Abstract

Adaptation and tolerance to changes in heat and cold temperature are essential for survival and proliferation in plants and animals. However, there is no clear information regarding the common molecules between animals and plants. In this study, we found that heat, and cold tolerance of the nematode *Caenorhabditis elegans* is oppositely regulated by the RNA-binding protein EMB-4, whose plant homolog contains polymorphism causing heat tolerance diversity. *Caenorhabditis elegans* alters its cold and heat tolerance depending on the previous cultivation temperature, wherein EMB-4 respectively acts as a positive and negative controller of heat and cold tolerance by altering gene expression. Among the genes whose expression is regulated by EMB-4, a phospholipid scramblase, and an acid sphingomyelinase, which are involved in membrane lipid metabolism, were found to play essential roles in the negative regulation of heat tolerance.

**Significance Statement**

Temperature is a critical environmental factor ever since the beginning of life and exerts a direct influence on biological activities in both plants and animals. Our study demonstrates that a homologous gene responsible for thermotolerance in plants, *emb-4*, is involved in the adaptation to temperature tolerance in the nematode *Caenorhabditis elegans*. The spliceosomal factor alters diverse gene expressions required for membrane lipid metabolism, which in turn regulates temperature tolerance.

## Introduction

All organisms on earth can survive by responding in appropriate ways to changing environmental factors such as temperature, humidity, light, and osmotic pressure. Temperature changes on a daily or seasonal basis, and strategies to cope with temperature changes are essential for the survival and proliferation of organisms. This is based on the processing of temperature information by the nervous system and the appropriate control of its downstream whole-body system (1–5). The nematode *Caenorhabditis elegans* is a useful model for studying temperature responses in animals because of its simple nervous system as well as powerful genetic tools (6). The temperature response of *C. elegans* has been investigated through various phenomena (7), such as cold tolerance (1, 8, 9), formation of dauer larvae resistant to high temperature (10), and thermotaxis, which is an associative learning between temperature and feeding state (11).

The cold tolerance of *C. elegans* is a phenomenon dependent on the cultivation temperature; for instance, adult worms grown at 25°C cannot survive at 2°C for 48 h, whereas worms grown at 15°C can survive at 2°C for 48 h (1, 12). Moreover, worms grown at 15°C lost cold tolerance after being transferred to 25°C for a few hours, a phenomenon termed as temperature acclimation (8, 9, 13, 14). When *C. elegans* acclimates to 25°C after a temperature change, it exhibits shortened lifespan, faster growth, movement, and more active energy expenditure. Conversely, worms acclimated to 15°C exhibit slower growth and moving and pumping activities (15–17). Cold tolerance is considered to be a result of the appropriate regulation of metabolic activities in the body due to acclimation to cultivation temperatures (1, 18, 19). We previously reported that the amount of fat in the gut is regulated downstream of the neural circuitry for temperature response as an important internal change involved in cold tolerance (20, 21). It is plausible that such metabolic changes caused by cultivation temperature affect not only cold tolerance but also the response to high temperatures.

The heat response of *C. elegans* has been extensively investigated. Heat shock stress induces the accumulation or misfolding of proteins; hence, organisms, including *C. elegans*, possess a genetically conserved program known as heat shock response (22). In experiments using *C. elegans*, acute heat shock at 33°C–37°C within a few hours is often used to induce a heat shock response (23, 24). Misfolded or unfolded proteins created by the heat shock are recognized and refolded by heat shock proteins and chaperones (25, 26). Lithgow et al. (27) reported that nonlethal high temperature stimulation as a pretreatment can enhance thermotolerance; in particular, wild-type N2 worms exposed to 30°C for 6 h could survive for a longer time during extreme heat stimuli at 35°C. Gouvea et al. (15) also reported that wild-type animals grown at 15°C, 20°C, and 25°C, respectively, show differences in reproduction activities after recovery with heat stress. Although it has been suggested that heat tolerance is modulated by previous environmental temperatures, the underlying molecular mechanism remains largely unknown.

High temperature also affects the development, photosynthesis, and yield of plants, causing oxidative stress, and drying; therefore, plants have the strategies to resist high temperatures through systematic changes in cellular and metabolic mechanisms (28, 29). *Arabidopsis thaliana* is a model organism with strong genetics and extensive global accessions that encompass phenotypic and genetic variations, and several responsible genes for thermotolerance have been reported (30–33). Isono et al. (34) identified *Long-term Heat Tolerance1* (*LHT1*) as a new gene involved in the natural variation of heat tolerance. The phenotypic differences between the two accessions of *A. thaliana*, of which *Col-0* accession is heat-sensitive and *Ms-0* accession is heat-tolerant, were due to polymorphisms in *LHT1/MAC7* (34). *LHT1/MAC7* gene encodes RNA helicase that is involved in mRNA splicing. *LHT1/MAC7* is homologous to Aquarius (AQR) in humans and *embryonic lethal* gene *emb-4* in *C. elegans*. Elucidating the thermotolerance mechanism involving *LHT1*/EMB-4 will provide new insights into the understanding of the thermotolerance mechanisms common to all organisms.

*emb-4* has been isolated as a gene essential for embryonic development (35), and the EMB-4 protein is primarily localized in germ cells and embryonic nuclei (36, 37). Tyc et al. (37) suggested that EMB-4 binds to the introns of target RNAs and cooperates with Argonaute proteins to ensure gene expression in the germline rather than to conduct splicing. They also reported that the loss of *emb-4* down-regulates the expression of genes involved in reproduction, chromatin modulation, and nuclear functions through transcriptome analysis in the absence of temperature stimuli (37). In fact, an *emb-4* mutant exhibits slow growth, embryonic or larval death, and defects in vulval development as well as abnormal gonad development (35). For instance, the null mutant *emb-4 (hc60)* is capable of producing sufficient adult progeny in 6 days when grown at 20°C; however, when it is grown at 15°C, the majority of eggs fail to hatch, and a very small number of eggs reach adulthood, indicating a cold-sensitive phenotype. To summarize, *emb-4* is a gene that exerts global effects on the gene expression program required for the developmental process, and a part of those effects are affected by the rearing temperature. On the basis of these previous findings, we hypothesized that the genes whose expression is regulated by EMB-4, the homolog of *LHT1* that determines high temperature tolerance in plants, are also involved in the temperature tolerance of *C. elegans.*

In this study, we found that the heat and cold tolerance of *C. elegans* is oppositely regulated by the intron binding protein AQR homolog EMB-4, whose plant homolog contains polymorphism causing heat tolerance diversity. EMB-4 might be involved in regulating the expression levels of stress response, metabolism, extracellular material, and noncoding RNA. In particular, a phospholipid scramblase, and an acid sphingomyelinase (ASM), which are involved in lipid metabolism, were involved in heat tolerance. Our findings indicate that lipid metabolism genes are involved in heat tolerance in a part of the downstream of EMB-4.

## Results and discussion

### Wild-type strain Bristol N2 exhibits heat tolerance dependent on cultivation temperature

**Fig. 1.**
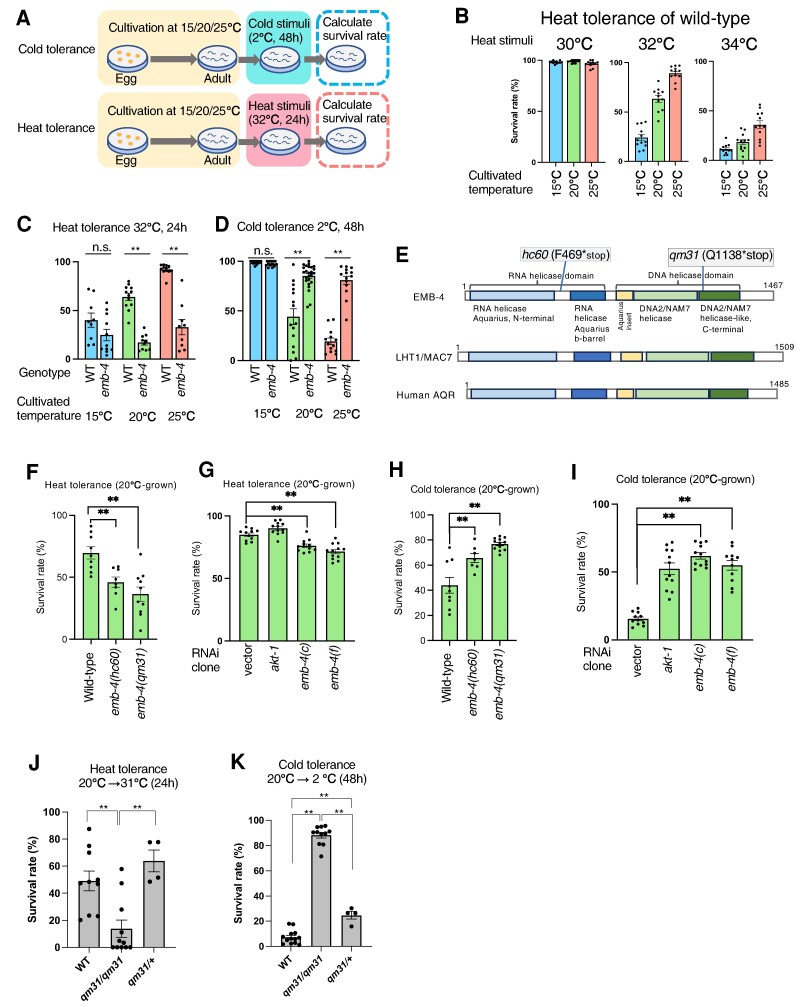
Heat and cold tolerance are oppositely regulated by EMB-4. A) Schematic illustration of cold tolerance and heat tolerance assays. In the cold tolerance assay, worms were cultivated at a constant temperature from egg to adult at 15°C or 20°C or 25°C, and then they were incubated at 2°C for 48 h, after which the survival rate was calculated the next day. In the heat tolerance assay also, worms were cultivated at a constant temperature and then incubated at 32°C for 24 h, after which the survival rate was calculated. B) Examination of heat temperature as a heat tolerance. Worms cultivated at 15°C, 20°C, and 25°C, respectively, were subjected to either 30°C, 32°C, or 34°C heat stimulation for 24 h. 30°C heat stimulation exerted no effect on survival, whereas 32°C and 34°C increased the survival as the rearing temperature increased. Number of assays >11 (*n* = 1,198, 814, and 642 worms for 30°C; *n* = 899, 971, and 1,207 worms for 32°C; *n* = 930, 918, and 1,160 worms for 34°C). C, D) *emb-4* mutant exhibited abnormal heat and cold tolerance. WT and *emb-4* as genotypes in the horizontal axis indicate wild-type and *emb-4(hc60)* mutant, respectively, and cultivation temperatures are indicated at bottom of the charts. When worms were cultivated at 20°C and 25°C, the survival rates of *emb-4* mutant in the heat tolerance assay were significantly lower than those of the wild-type strain, but in the cold tolerance assay, its survival rates were significantly higher than those of the wild-type strain. Number of assays ≥9 (*n* = 382, 511, 662, 479, 918, and 444 worms). E) Schematic illustration of EMB-4, LHT1/MAC7, and AQR proteins. *Caenorhabditis elegans* EMB-4, plant LHT1/MAC7, and human AQR all have a RNA helicase domain, a DNA helicase domain, and a linker-domain that links them together. These domain structures were predicted by a database, InterPro 99.0 EMBL-EBI (https://www.ebi.ac.UK/interpro/). F, H) Both *emb-4(hc60)* and *emb-4(qm31)* mutants exhibited similar abnormalities in the cold and heat tolerance assays. Number of assays ≥ 7 ([F] *n* = 629, 243, and 486 worms, [H] *n* = 639, 307, and 703 worms). G, I) RNA interference of *emb-4* caused weakly abnormal phenotypes in the cold and heat tolerance assays, which were consistent with the phenotypes of *emb-4* null mutants. Number of assays ≥ 11 ([G] *n* = 680, 760, 723, and 628 worms, [I] *n* = 708, 704, 623, and 538 worms). J) A heterozygous animal with the maternal effect mutation *emb-4(qm31)* demonstrated normal heat tolerance. All the animals were grown at 20°C. Number of assays ≥ 4 (*n* = 660, 246, and 216 worms). K) Cold tolerance in the *emb-4(qm31)* heterozygote. All the animals were grown at 20°C. Number of assays ≥ 4 (*n* = 920, 356, and 216 worms). The error bars indicate SEMs. n.s. *P* ≥ 0.05; *P < 0.05; **P < 0.01. Comparisons were performed using Welch's *t* tests (C, D) or one-way ANOVA followed by Dunnett's post hoc tests (F–I), or followed by Tukey Kramer's post hoc tests (J, K).

We have reported that the wild-type strain N2 gains or loses cold tolerance dependent on its previous cultivation temperature (Fig. 1A). Here, we evaluated the dependency of heat tolerance on its previous cultivation temperature. For instance, wild-type worms were cultivated at 15°C, 20°C, and 25°C, respectively, and then exposed to high temperature stimulation for 24 h at 30°C, 32°C, and 34°C, after which their survival rates were calculated (Fig. 1B).

The worms cultivated at 15°C, 20°C, or 25°C were alive after exposure to the heat stimulus of 30°C for 24 h (Fig. 1B), suggesting that 30°C is not a sufficiently high temperature to kill the wild-type worms irrespective of the previous cultivation temperature. However, the majority of wild-type worms cultivated at 15°C died at 32°C, whereas those cultivated at 25°C were alive at 32°C, and worms cultivated at 20°C exhibited their intermediate phenotype (Fig. 1B, C). Similarly, approximately 36% of 25°C-cultivated wild-type worms were alive, whereas worms cultivated at 15°C and 20°C died at 34°C (Fig. 1B). These results indicated that wild-type worms cultivated at higher temperatures exhibited higher survival rates after exposure to 32°C or 34°C, implying that gaining, or losing heat tolerance depends on the cultivation temperature. This finding is consistent with previous reports on tolerance to high temperatures (15).

We investigated the cause of the difference in survival rates between 15°C- and 25°C-grown worms after exposure to heat for 24 h, because it is suggested that cultivation temperature information directly promotes systemic changes that can result in the gain or loss of heat tolerance.

### *emb-4* is responsible for abnormalities in heat tolerance and cold tolerance

The wild-type *C. elegans* strain N2 exhibited heat tolerance in an incubation temperature-dependent manner (Fig. 1B). Isono et al. (34) recently found that the gene responsible for heat tolerance in the natural accession of *A. thaliana* is *LHT1* that encodes a spliceosome factor. *emb-4* in *C. elegans* and *AQR* in humans are a homolog of *LHT1*, with similar protein structures of these three gene products that include an RNA helicase domain, a DNA2/NAM7 helicase domain, and a linker-domain that connects them together (Fig. 1E). We then investigated heat, and cold tolerance of *C. elegans emb-4* mutant after cultivation at different temperatures. *Caenorhabditis elegans emb-4* had been identified as a critical gene for embryonic development (35, 38), and Tyc et al. (37) suggested that EMB-4 functions as an intron binding protein that is involved in germline gene expression with Argonaute proteins.

We examined the heat tolerance of wild-type N2 and *emb-4(hc60)* null mutants after cultivation at 15°C, 20°C, or 25°C and then exposure to 32°C for 24 h. The 20°C- and 25°C-cultivated *emb-4(hc60)* mutant worms exhibited significantly lower heat tolerance than the wild-type strain (Fig. 1C), whereas the 15°C-cultivated *emb-4(hc60)* mutant worms exhibited a similar phenotype to that of the wild-type strain (Fig. 1C). Similarly, another mutation of *emb-4*, *emb-4(qm31)*, caused abnormal heat tolerance as that in the *emb-4(hc60)* mutant (Fig. 1F), and RNAi knockdown of *emb-4* reduced the heat tolerance, although the effect of RNAi knockdown was weaker than that of the genetic knockout of *emb-4* (Fig. 1G).

We next investigated the cold tolerance of *emb-4(hc60)* mutants after cultivation at 15°C, 20°C, or 25°C and then exposure to 2°C for 48 h. The 20°C- and 25°C-cultivated *emb-4(hc60)* mutants exhibited significantly higher cold tolerance than the wild-type strain (Fig. 1D), whereas the 15°C-cultivated *emb-4(hc60)* mutants exhibited a similar phenotype to that of the wild-type strain (Fig. 1D). Similarly, *emb-4(qm31)* caused abnormal increased cold tolerance as that of *emb-4(hc60)* (Fig. 1H), and RNAi knockdown of *emb-4* increased the cold tolerance (Fig. 1I). These data indicate that EMB-4 negatively regulates cold tolerance after cultivation at 20°C and 25°C.

### Heterozygote of maternal effect allele *emb-4(qm31)* exhibited wild-type-like heat and cold tolerance

Since the *emb-4* gene is expressed predominantly in germ cells and embryonic nuclei (37), we investigated whether the abnormal heat and cold tolerance of the *emb-4* mutant is due to its function in germ cells or not. We used the maternal effect allele *emb-4(qm31)*, which has similar phenotypes to *emb-4(hc60)* in terms of heat and cold tolerance (Fig. 1F, H). Because the maternal effect is thought to be delivered by maternal proteins or mRNA, *emb-4(qm31)* hermaphrodites were crossed to wild-type N2 males, and the phenotypes of *emb-4(qm31)/+* animals were studied (Fig. 1J, K).

*emb-4(qm31)* homozygous animals (*qm31/qm31*) showed significantly decreased heat tolerance after being grown at 20°C, whereas *emb-4(qm31)/+* heterozygous animals showed similar heat-tolerant phenotype as wild-type (+/+) (Fig. 1J). Also, *emb-4(qm31)* homozygous animals (*qm31/qm31*) showed significantly increased cold tolerance at 2°C after being grown at 20°C, whereas *emb-4(qm31)/+* heterozygous animals died at 2°C, which is similar to the phenotype of wild-type (+/+) animals (Fig. 1K), although *qm31/+* animals slightly showed increased cold tolerance than wild-type. Namely, disrupting EMB-4 only in early development did not strongly affect heat and cold tolerance. These genetic analyses indicate that heat and cold tolerance are regulated either by EMB-4 at a site of action outside of germ cells or by some as yet undiscovered function of the EMB-4 protein within germ cells during the late larval stage or adult stage.

### Gene expression is altered by the loss of *emb-4* under heat or cold stimulation

A previous report suggested that EMB-4 plays an essential role in germ cell gene expression and regulation (37). Tyc et al. reported that there are approximately 7,000 genes differentially expressed in *emb-4(hc60)* mutant relative to the wild-type strain in the absence of temperature stimuli. To explore the changes in gene expression levels between *emb-4* mutant and wild-type strains exposed to cold and heat stress, we conducted transcriptome analyses under various temperature conditions.

We investigated the appropriate temperature shift conditions for the isolation of mRNA from the worm during the process of losing cold and heat tolerance. To isolate mRNA from the worms just before their death under cold or heat stress, we determined the threshold time for death under cold or heat stress. The 20°C-cultivated wild-type and *emb-4* mutant worms survived when exposed to 2°C for 9 h (Fig. S1A), but they were dead when exposed to 2°C for 18 h. Hence, we used “2°C for 9 h” as a condition for the isolation of mRNA from the worms during the process of losing cold tolerance.

Wild-type worms died when exposed to 32°C for 24 h (Fig. S1B), but they survived when exposed to 32°C for 13 h. Therefore, we used “32°C for 13 h” as a condition for isolating mRNA from the worms during the process of losing heat tolerance. Because it has been reported that 1-h heat shock induces gene expression, such as classical heat shock proteins, we also used “32°C for 1 h” as a condition for understanding rapid heat responses via EMB-4.

**Fig. 2.**
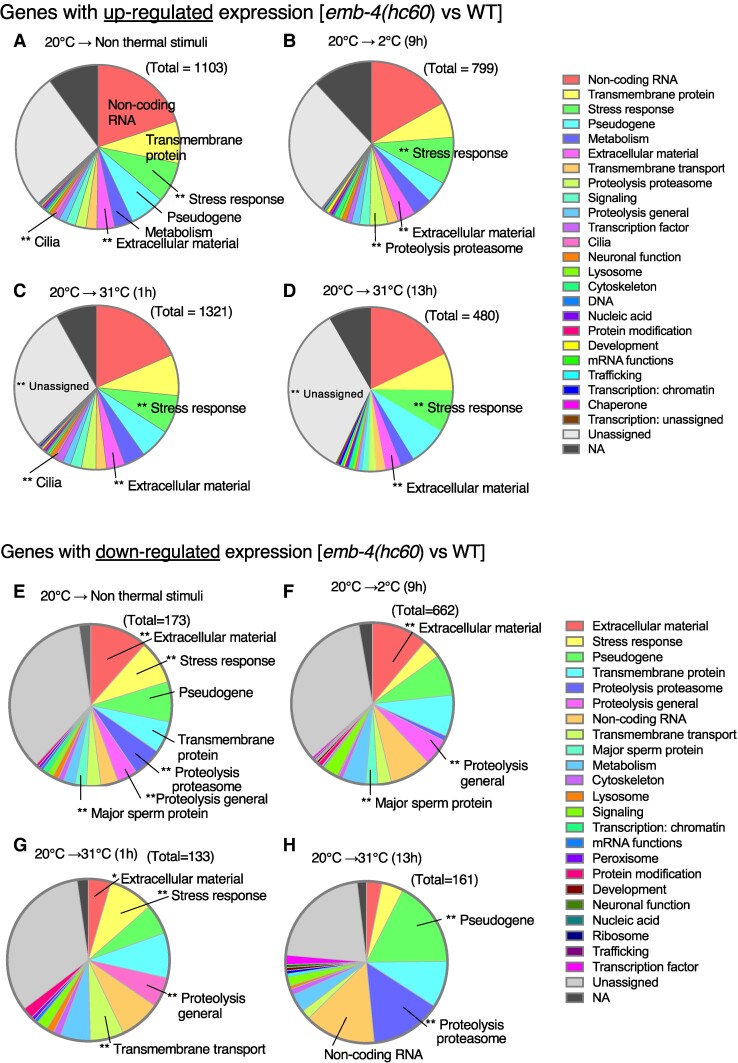
Genes whose expression was up- or down-regulated by the loss of *emb-4*. RNA-Seq analysis identified differentially expressed genes in *emb-4(hc60)* mutant compared with wild-type adult worms using DESeq2. Genes were classified as upregulated by log_2_FC > 1 and FDR *P*-value of <0.05, and genes were classified as down-regulated by log_2_FC < -1 and FDR *P*-value of <0.05. Gene ontology enrichment analyses were performed in WormCat (52). Triple replicates of total RNA samples were analyzed per genotype and temperature condition. Temperature conditions used were 20°C-grown (A, E), 20°C-grown and then incubation at 2°C for 9 h (B, F), 20°C-grown and then incubation at 31°C for 1 h (C, G), and 20°C-grown and then incubation at 31°C for 13 h (D, H). The total in the figure is the number of upregulated or down-regulated genes of each comparison. Gene ontology categories are indicated with the color key in the right panel. The number of genes enriched in individual GO terms analyzed by Wormcat 2.0 are listed in the Data S1 Up_rgs_categories.xlsx and Data S2 Down_rgs_categories.xlsx.

The differences in gene expression between *emb-4(hc60)* mutant and wild-type strains were determined under four conditions by transcriptome analyses. We detected 1,103 genes whose expression levels were upregulated when compared between *emb-4(hc60)* mutant and wild-type strains after cultivation at 20°C without a temperature stimulus (20°C → Non thermal stimuli) (Fig. 2A). Among these genes, the gene classes such as stress response, extracellular material, and cilia were significantly more abundant according to gene ontology analysis (Fig. 2A). After cultivation at 20°C followed by incubation at 2°C for 9 h (20°C → 2°C [9 h]), when the worms tolerate cold stimuli without dying, the expression levels of 799 genes were upregulated in *emb-4(hc60)* mutants compared with those in the wild-type strain. After cultivation at 20°C followed by incubation at 32°C for 1 h (20°C → 31°C [1 h]) or 13 h (20°C → 31°C [13 h]), the expression levels of 1,321 or 480 genes were respectively upregulated in *emb-4(hc60)* mutants compared with those in the wild-type strain. Among these genes, those related to stress response were upregulated under all four conditions (Fig. 2A–D). In all four tested conditions (20°C → Non thermal stimuli, 2°C [9 h], 31°C [1 h], or 31°C [13 h]), the expression levels of noncoding RNAs were upregulated in the *emb-4* mutant compared to the wild-type (Fig. 2A–D), implying that EMB-4 may be involved in the expression of noncoding RNA, either indirectly or directly.

Similarly, we identified the genes whose expression levels were down-regulated when compared between *emb-4(hc60)* mutant and wild-type strains under the four types of temperature shift protocols, viz., (20°C → non thermal stimuli), (20°C → 2°C [9 h]), (20°C → 32°C [1 h]), and (20°C → 32°C [13 h]), as shown in Fig. 2E–H, respectively. Using the four protocols, we respectively identified 173, 662, 133, and 161 genes whose expression levels were down-regulated when compared between *emb-4(hc60)* mutant and wild-type strains (Fig. 2E–H). Among them, the genes related to proteolysis were down-regulated under all four conditions (Fig. 2E–H).

**Fig. 3.**
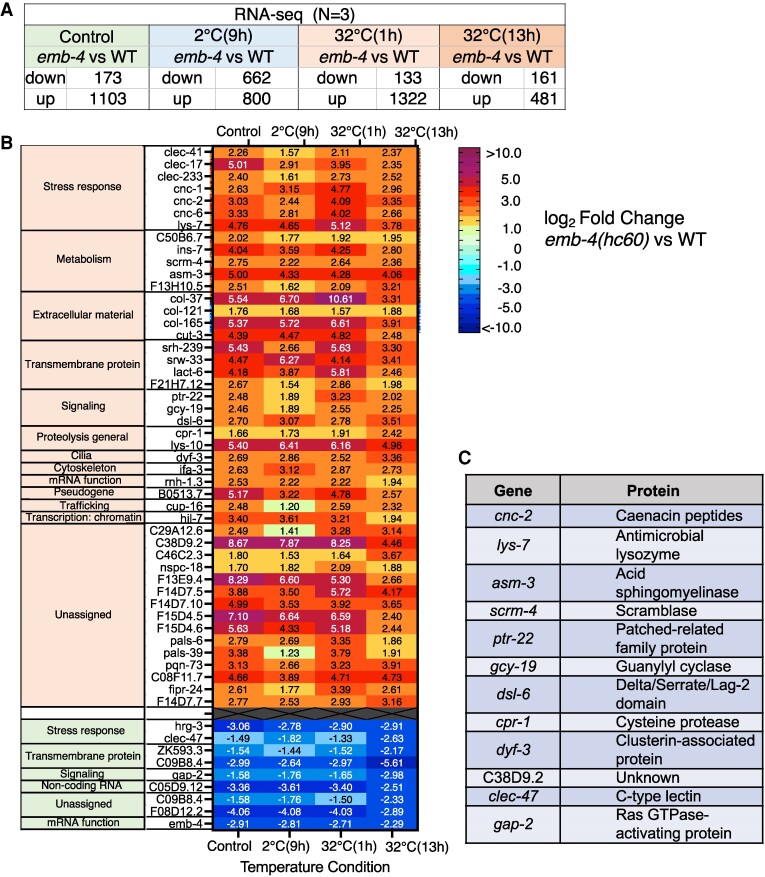
Genes whose expression was regulated by EMB-4 irrespective of temperature stimuli. A) Number of genes whose expression was upregulated or down-regulated in the four temperature shift protocols. B) Heat map of 55 gene expression levels that were commonly upregulated or down-regulated in all protocols (Fig. 4B). Forty-six of the genes were upregulated, and nine were down-regulated. Color bars depicting relative expression levels (log_2_FC *emb-4*/WT) are shown on the right. C) Among the 55 genes, the expression of 12 genes was affected by *emb-4*, and mutants had already been isolated and used for phenotypic analysis of temperature tolerance.

We compiled a list of genes whose expression was up- or down-regulated in the four temperature shift protocols mentioned above (Fig. 3A) and identified 55 genes whose expression varied consistently across all four protocols (Fig. 3B). Of these 55 genes, 46 were upregulated in the *emb-4* mutant, while nine were down-regulated, including several genes related to stress response and metabolism.

### Analysis of the involvement of genes whose expression is affected by EMB-4 in cold tolerance

**Fig. 4.**
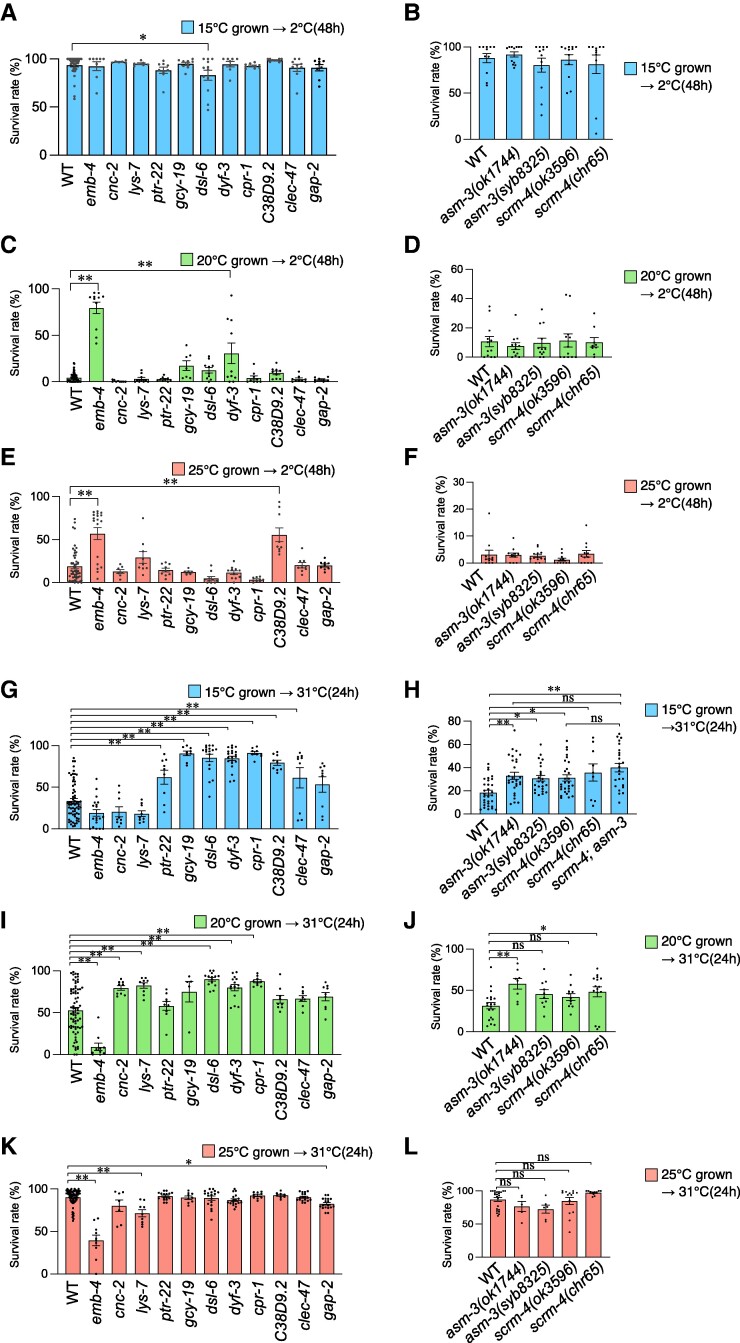
Several genes whose expression was affected by *emb-4* were involved in cold or heat tolerance. A–F) The survival rate in the cold tolerance assay with 12 gene mutants whose expressions were upregulated or down-regulated by the loss of *emb-4*. Worms were cultivated at the designated temperatures (at 15°C [A, B], 20°C [C, D], and 25°C [E, F]) constantly from egg to adult, and then they were transferred and incubated at 2°C for 48 h, after which the survival rates were calculated. Number of assays ≥ 6. ([A] *n* = 3215, 154, 668, 1,129, 590, 467, 303, 351, 730, 706, 593, and 574 worms, [B] *n* = 597, 636, 610, 749, and 691 worms, [C] *n* = 3721, 249, 400, 367, 428, 261, 816, 776, 447, 380, 507, and 374 worms, [D] *n* = 758, 708, 824, 851, and 849 worms, [E] *n* = 3,257, 823, 567, 558, 478, 283, 347, 402, 823, 516, 431, 500, and 406 worms, [F] *n* = 735, 676, 811, 690, and 775 worms). G–L) The survival rate in the heat tolerance assay with 12 mutants whose expressions were upregulated or down-regulated by the loss of *emb-4*. Worms were cultivated at the designated temperatures (at 15°C [G, H], 20°C [I, J], and 25°C [K, L]) constantly from egg to adult, and then they were transferred and incubated at 31°C for 24 h, after which the survival rates were calculated. Number of assays ≥ 5. ([G] *n* = 4,371, 746, 1,217, 1,189, 481, 548, 911, 1,066, 659, 685, 502, and 477 worms, [H] *n* = 2,052, 2,108, 1,845, 2,580, 1,210, and 1,833 worms, [I] *n* = 3,727, 322, 861, 673, 393, 268, 699, 731, 442, 447, 402, and 410 worms, [J] *n* = 932, 420, 338, 846, and 985 worms, [K] *n* = 3,376, 300, 517, 493, 766, 458, 912, 808, 578, 385, 932, and 954 worms, [L] *n* = 1305, 225, 248, 921, and 648 worms). The error bars indicate SEMs. n.s. *P* ≥ 0.05; *P < 0.05; **P < 0.01. Comparisons were performed using one-way ANOVA followed by Dunnett's post hoc tests (A–G, I–L), or followed by Tukey Kramer's post hoc tests (H).

We considered that an expression change(s) in a part of the 55 genes may be related to abnormalities in the heat or cold tolerance of *emb-4* mutants. As mutations in 12 of the 55 genes have been isolated previously, we examined the heat and cold tolerance of these mutants (Figs. 3C and 4). In the *emb-4* mutant, we observed upregulated gene expressions in 10 of the 12 genes, viz., *cnc-2*, *lys-7*, *asm-3*, *scrm-4*, *ptr-22*, *gcy-19*, *dsl-6*, *cpr-1*, *dyf-3*, and *C38D9.2* (Fig. 3B). We also detected down-regulated gene expressions in 2 of the 12 genes, viz., *clec-47* and *gap-2* (Fig. 3B).

We evaluated the cold tolerance of those 12 gene mutants after cultivation at 15°C (Fig. 4A, B), 20°C (Fig. 4C, D), and 25°C (Fig. 4E, F), respectively. The *dsl-6* mutant with an impaired Notch binding protein exhibited slightly abnormal decreased cold tolerance after cultivation at 15°C (Fig. 4A). We found that *dyf-3* mutant exhibited slightly abnormal increased cold tolerance after cultivation at 20°C (Fig. 4C). *dyf-3* encodes clusterin-associated protein that mediates development of cilia structure of sensory neurons in the nervous system (39), suggesting that DYF-3 may cause abnormal cilia structure of a neuron(s) required for cold tolerance. In the cold tolerance after cultivation at 25°C, we found that the novel gene C38D9.2 mutant worms displayed high survival rates (Fig. 4E).

### Some genes whose expression is affected by EMB-4 are involved in heat tolerance

We also evaluated the heat tolerance of the 12 gene mutants (Fig. 3C) after cultivation at 15°C (Fig. 4G, H), 20°C (Fig. 4I, J), and 25°C (Fig. 4K, L), respectively. We found that the nine mutants of *asm-3*, *scrm-4*, *ptr-22*, *gcy-19*, *dsl-6*, *dyf-3*, *cpr-1*, *C38D9.2*, and *clec-47* exhibited increased heat tolerance after cultivation at 15°C (Figs. 4G, H and S1C). *scrm-4* encodes scramblase, and *asm-3* encodes ASM; these two genes are involved in lipid metabolism, implying that lipid metabolism regulates heat tolerance after cultivation at 15°C. The *cnc-2*, *lys-7, dsl-6*, *dyf-3*, and *cpr-1* mutants exhibited increased heat tolerance after cultivation at 20°C (Fig. 4I). One of the two null mutations in the *scrm-4* and *asm-3* genes resulted in slightly increased heat tolerance after cultivation at 20°C, implying that *scrm-4* and *asm-3* are not involved in heat tolerance after cultivation at 20°C. Alternatively, these genes may play a minor role in the phenomenon after cultivation at 20°C (Fig. 4J). We detected that the two mutants of *lys-7* and *gap-2* exhibited decreased heat tolerance after cultivation at 25°C (Fig. 4K).

The *dsl-6*, *dyf-3*, and *cpr-1* mutant with an impaired notch-delta signaling, neuronal cilial component, and cysteine protease exhibited abnormal increased heat tolerance after cultivation at 15°C and 20°C, suggesting that these gene products negatively regulate heat tolerance at any cultivation temperature (Fig. 4G, I).

The *lys-7* mutant with an impaired lysozyme involved in defense response to other organism exhibited increased heat tolerance after cultivation at 20°C (Fig. 4I). However, this mutant worms exhibited decreased heat tolerance after cultivation at 25°C (Fig. 4K), indicating that LYS-7 regulate heat tolerance after cultivation at 20°C and 25°C in an opposite direction.

### *emb-4* is genetically downstream of *asm-3* and upstream of *scrm-4*

**Fig. 5.**
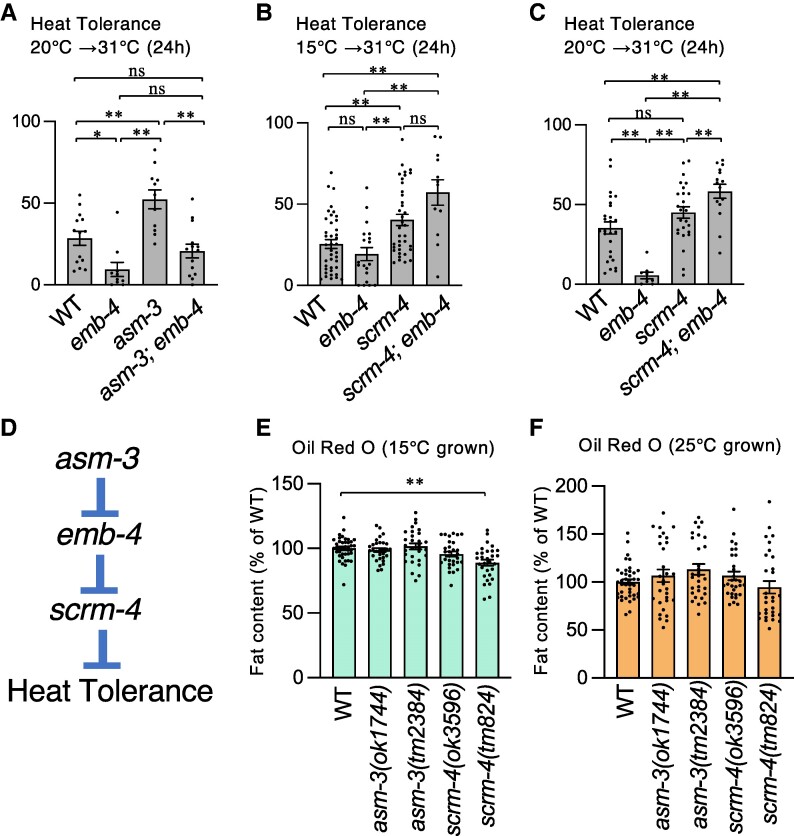
Lipid metabolism-related genes were involved in heat tolerance. A–C) Survival rate in the heat tolerance assay with a double mutant that impairs lipid metabolism-related genes *asm-3* and *scrm-4*. Worms were grown at the specified temperatures (at 20°C [A, C] and 15°C [B]) constantly from egg to adult, then transferred and incubated at 31°C for 24 h, and survival rates were calculated. Number of assays ≥ 10. ([A] *n* = 662, 322, 551, and 440 worms, [B] *n* = 2,798, 746, 3,320, and 284 worms, [C] *n* = 1,371, 322, 1,862, and 457 worms). D) A schematic diagram of the genetic pathway for heat tolerance. Genetic epistasis analysis of mutations in *emb-4*, *scrm-4* lacking scramblase, and *asm-3* lacking acid sphingomyelinase (ASM) suggested a genetic model for heat tolerance in which *emb*-4 acts at downstream of *asm*-3, *scrm-4* acts at downstream of EMB-4, and *scrm-4* and *asm-3* act in the same genetic pathway. E, F) Fat content in the gut was measured by oil red O staining. Worms were cultivated at 15°C (E) or 25°C (F) from egg to adult, and then day-1 adult worms were fixed and stained with oil red O. The fat content value in each bar is a relative value to the average value of wild-type worms. ([E] *n* = 40, 30, 30, 30, and 30 worms, [F] *n* = 40, 30, 30, 30, and 30 worms). The error bars indicate SEMs. n.s. *P* ≥ 0.05; *P < 0.05; **P < 0.01. The comparisons were performed using one-way ANOVA followed by Tukey Kramer's post hoc tests (A–C), or followed by Dunnett's post hoc tests (E, F).

Previous studies have shown that fat metabolism and fatty acid composition control are critical for cold tolerance and temperature acclimation in *C. elegans* (1, 20, 21); however, no correlation between heat tolerance and fat content has been reported. Therefore, we focused on heat tolerance by targeting the *asm-3* gene, which encodes ASM, and the *scrm-4* gene, which encodes phospholipid scramblase, both of which are involved in lipid metabolism. Heat tolerance in the *asm-3* and *scrm-4* mutants was higher than in the wild-type strain (Figs. 4H and S1C), indicating that abnormally elevated gene expression of the *asm-3* and *scrm-4* genes resulted in abnormal heat tolerance in the *emb-4* mutant (Fig. 3B). However, the increased gene expression of *scrm-4* and *asm-3* genes in the *emb-4* mutant does not distinguish between the two regulatory mechanisms: these genes are either simply downstream of EMB-4 or serve as gene compensation for the down-regulation of these genes in the *emb-4* mutant. To better understand the causal relationship between these genes, we created the double mutants—*asm-3; emb-4, scrm-4; emb-4*, and *scrm-4; asm-3*—to determine whether the heat tolerance phenotypes of each single mutant are enhanced, suppressed, or nearly identical to those of either single mutation (Figs. 4H and 5A–C).

The *asm-3; emb-4* double mutant had a similar heat tolerance phenotype to the *emb-4* single mutant, and the abnormally increased heat tolerance caused by the *asm-3* mutation was suppressed by the *emb-4* mutation (Fig. 5A). Specifically, the *emb-4* mutation is epistatic to the *asm-3* mutation, indicating that EMB-4 acts downstream of ASM-3, in heat tolerance regulation (Fig. 5D). The *scrm-4; emb-4* double mutant exhibited the similar heat tolerance phenotype as the *scrm-4* single mutant (Fig. 5B, C), indicating that the *emb-4* mutation was suppressed by the *scrm-4* mutation. This suggests that the *scrm-4* mutation is epistatic to the *emb-4* mutation, with SCRM-4 acting downstream of EMB-4 (Fig. 5D). We discovered that the *scrm-4; asm-3* double mutant exhibited similar heat tolerance phenotypes to either single mutant (Fig. 4H). This genetic interaction indicates that *scrm-4* and *asm-3* share the same genetic pathway (Fig. 5D).

### Acid sphingomyelinase ASM-3 and phospholipid scramblase SCRM-4 are negative regulators of heat tolerance

In the *emb-4* mutant, the expression of *asm-3* encoding ASM was constitutively upregulated (Fig. 3B), and heat tolerance was decreased compared with that in the wild-type strain (Fig. 1B). However, impaired *asm-3* caused abnormal increased heat tolerance, observed in three *asm-3(ok1744), asm-3(syb8325)*, and *asm-3(tm2384)* mutants (Figs. 4H and S1C). Therefore, ASM-3 negatively regulates heat tolerance. ASM is generally located in the lipid bilayer such as the plasma membrane or the lysosome, in which ASM hydrolyzes sphingomyelin to produce ceramide and phosphocholine (40, 41). At the cellular level, sphingolipids, including ceramide, produced by ASM are important molecules involved in the organization of the lipid raft, which is an integral center of several biological processes in cellular membrane signaling (42). At the individual level, the lifespan of *C. elegans* is extended in the *asm-3* mutant, which is caused by abnormal fatty acid metabolism, wherein an excessive accumulation of two types of omega-3 long-chain fatty acids occurs (41, 43). Altogether, it is hypothesized that ASM-3-dependent cellular membrane signaling and/or ASM-3-dependent fatty acid metabolism plays a key role in controlling heat tolerance.

In the *emb-4* mutant, the expression of *scrm-4* was also constitutively upregulated (Fig. 3B), and heat tolerance was decreased compared with that in the wild-type strain (Fig. 1B). Impaired *scrm-4* expression caused abnormal increased heat tolerance, which was observed in the three types of *scrm-4* knockout mutants (Figs. 4H and S1C). These results suggest that SCRM-4 exerts an inhibitory effect on heat tolerance. A phospholipid scramblase (SCRM) is also generally located in the lipid bilayer such as the plasma membrane or mitochondria, in which scramblase externalizes phosphatidylserine from the inner membrane to the outer membrane (44). The major activator of scramblase is calcium ions, which are induced by blood coagulation, apoptosis, and mitophagy signaling (44, 45). A recent report suggested that one of the phospholipid scramblases, ATG-9, is involved in lipid mobilization from lipid droplets in *C. elegans* (46). Together with these results and knowledge, it is hypothesized that a local composition of membrane phospholipids regulated by calcium ions could control SCRM-4-dependent heat tolerance in *C. elegans*.

We have previously reported that a correlation exists between gut fat content and cold tolerance, with increased gut fat content allowing survival at 2°C, but not at 2°C with decreased gut fat content (20). A previous study suggested that the neutral fat content in the intestine of 15°C-cultivated wild-type animals is approximately two times higher than that of 25°C-grown animals (20), which is a reason for survival at 2°C in the 15°C-cultivated wild-type strain (Fig. 1D). Conversely, 25°C-cultivated wild-type worms could tolerate heat stimulus such as 31°C for 24 h (Fig. 1C). The correlation between heat tolerance and fat content has not been reported. We examined whether *asm-3* or *scrm-4* mutations affect the amount of fat in the intestine by oil-red-O staining, which detects neutral lipids included in the lipid droplets. The fat content of *asm-3* mutant was similar to that of the wild-type strain, but that of *scrm-4(tm624)* mutant was slightly less than that of the wild-type strain after cultivation at 15°C (Fig. 5E). After cultivation at 25°C, the fat content of *asm-3* and *scrm-4* mutants was similar to that of the wild-type strain (Fig. 5F). These findings suggest that a phospholipid scramblase, SCRM-4, could be involved in fat accumulation in the gut after cultivation at 15°C, and that the increasing heat tolerance did not necessarily correlate with the neutral fat content in the gut.

In this study, our results suggested that cultivation temperature–dependent heat and cold tolerance are affected by the loss of the intron binding protein EMB-4 conserved between *C. elegans* and plants. The absence of EMB-4 alters the transcriptional profiles of several genes, implying that abnormalities of heat and cold tolerance in *emb-4* mutants are due to a combination of multiple factors. After incubation at 15°C, the wild-type worm gains cold tolerance and loses heat tolerance, which is an adaptive mechanism and an advantage for survival at 15°C in nature. Adaptive mechanisms, allowing for such tradeoffs, would be properly regulated by a nervous system. However, the systemic control mechanisms remain to be elucidated. Further investigation of the molecular pathways and upstream factors of phospholipid metabolism involving ASM and SCRM with EMB-4 will help discover the unresolved mechanisms of adaptation to the environment in living organisms.

## Materials and methods

### Ethical issues and approval

All animal treatments in this research were performed according to the Japanese Act on Welfare and Management of Animals (Act No. 105 of 1973 October 1; latest revisions Act No. 51 of 2017 June 2, Effective 2018 June 1). All experimental protocols were approved by the Institutional Animal Care and Use Committees of Konan University.

### Strains

*Caenorhabditis elegans* was grown under standard conditions (47). The N2 strain was used as the wild-type strain in all experiments. The following strains were used: MJ60 *emb-4(hc60)*, MQ464 *emb-4(qm31)*, *eri-1(mg366);*l*in-15B(n744)*, RB2374 *cnc-2(ok3226)*, CB6738 *lys-7(ok1384)*, *RB1487 asm-3(ok1744)*, *asm-3(tm2384), asm-3(syb8325)*, *RB2584 scrm-4(ok3596)*, *scrm-4(tm624)*, *scrm-4(chr65),* VC4139 *ptr-22(gk5222)*, *RB1909 gcy-19(ok2472)*, *RB1762 dsl-6(ok2265)*, *cpr-1(ok1344)*, *SP1603 dyf-3(m185)*, *RB1543 C38D9.2(ok1853)*, PS8825 *clec-47(sy1532)*, and JN147 *gap-2(tm748)*, *scrm-4(ok3596); asm-3(ok1744), asm-3(ok1744); emb-4(qm31), scrm-4(ok3596); emb-4(qm31)*. We outcrossed the *asm-3(ok1744)* and *scrm-4(ok3596)* mutations two and three times, respectively with the wild-type N2 strain in this study. *scrm-4(chr65)*, *asm-3(syb8325)*, *scrm-4(ok3596); asm-3(ok1744), asm-3(ok1744); emb-4(qm31), scrm-4(ok3596); emb-4(qm31)* mutant strains were constructed in this study. *emb-4(qm31)*/+ heterozygous hermaphrodites were obtained by crossing *emb-4(qm31)* homozygous hermaphrodites with wild-type N2 males.

### Cold tolerance assay

The cold tolerance assay was performed using a previously reported procedure (1, 13). In this assay, well-fed adult worms were placed on nematode growth medium (NGM) containing 2% (w/v) agar seeded with *Escherichia coli* OP50 on the medium. The adult worms were cultivated for 16–24 h at 15°C to lay eggs, and the adult animals were removed afterward. The progenies were left to mature at 15°C for 120–130 h. Well-fed adults were placed on the plates to lay eggs, the adults were removed after 6–15 h at 20°C or 25°C, and the progeny were left to mature at 20°C for 72–80 h or 25°C for 60–72 h. However, as the *emb-4* mutant has a high frequency of developmental abnormalities at 15°C or 25°C, the eggs were raised at 20°C for 6 days until reaching the L4 larval stage and then grown at 15°C, 20°C, and 25°C for at least 24 h to reach the adult stage, which were termed as 15°C-, 20°C-, and 25°C-grown worms, respectively. Before the next generation of worms hatched, the plates containing fresh adults were placed on ice for 20 min and then transferred to a 2°C refrigerated cabinet (CDB-14A; DAIWA Industries LTD, Japan) for 48 h; the temperature inside this cabinet was monitored using a digital thermometer. After cold stimulation, the plates were stored at 15°C overnight. Live and dead worms were counted, and survival rates were calculated.

### Heat tolerance assay

The rearing conditions were exactly similar to those described for the cold tolerance assay. Test plates with only fresh adults reared at 15°C, 20°C, and 25°C were transferred into the peltier-type compact incubator (CN-25C; Mitsubishi, Japan) set at 30, 31, 32, or 34°C or the cabinet (CDB-14A; DAIWA Industries LTD, Japan) set at 31°C and then incubated for 24 h; the temperature of the every shelf inside these incubators was monitored using a wireless digital thermometer (SaverisH3D; Testo, Germany). Next, the plates were stored at 15°C overnight. Live and dead worms were counted, and survival rates were calculated. When we conducted the analyses for Fig. 4H in the winter season, the heat tolerance of wild-type animals decreased compared to the results shown in Fig. 4G, where the analyses were conducted in the rainy seasons; thus, this observed difference could have been caused by humidity and other unknown factors, as described previously in temperature acclimation and cold tolerance (5, 13, 18).

### RNA interference

The feeding RNAi protocol was performed as described in previous reports (48, 49). The *C. elegans* RNA interference (RNAi) library, which had been established by Ahringer, was purchased from source BioScience (50) and used for all RNAi analyses in this study. The following RNAi clones were used: L4440 empty vector control, *akt-1 (*clone C12D8.10), *dpy-11 (*clone F46E10.9*)*, and *emb-4* (clone Y80D3A.c and Y80D3A.f). We used *eri-1 (mg366); lin-15B (n744)*, which was a neuronal RNAi-sensitive strain in feeding RNAi (49). *akt-1 (RNAi)*, which impairs AKT kinase in insulin signaling, was used as a positive control for cold tolerance as reported previously (18). The plate for feeding RNAi contained NGM agar supplemented with 100 µg/mL ampicillin and 0.1 mM isopropyl-β-D-thiogalactopyranoside (IPTG). *Escherichia coli* HT115 carrying the specific RNAi clones were cultured in 2 mL of LB liquid medium containing 100 µg/mL ampicillin for 5 h, and then the culture was centrifuged, the pellet was resuspended with 600 µL of LB medium containing 100 µg/mL ampicillin and 0.12 mM IPTG. Some drops of this suspension were applied to NGM and left overnight at room temperature, which was used as the feeding RNAi plate. L4 stage worms were placed on the feeding RNAi plate, and after incubation for 24 h at 20°C, the adults were moved to a new feeding RNAi plate and left for another 24 h to lay eggs. The eggs were left to mature at 20°C for 72–80 h. Because of developmental abnormalities due to the knockdown of *emb-4*, *emb-4* knocked down worms were allowed to mature for 96–115 h. Adult worms were used for cold or heat tolerance assay.

### RNA-Seq analysis

Worms were cultivated at 20°C until reaching the adult stage, and then they were collected immediately after a designated period of exposure to cold or heat stimuli. Worms were washed three times with M9 buffer, and the settled worms were mixed with the homogenization solution provided in the Maxwell RSC Tissue RNA kit (Promega, #AS1340). The worms dissolved in the homogenization solution were frozen in liquid nitrogen, immediately thawed, and crushed with 5.0-mm metal beads and 0.6-mm silica beads (Biomedical Science, Japan) in a bead crusher (FastPrep 24, MP-Biomedicals). Total RNA was extracted from the crushed worms in the homogenization solution using the RSC Tissue RNA kit and Maxwell RSC instrument (Promega). RNA-seq libraries were prepared using TruSeq RNA sample prep kit v2 (Illumina) according to the manufacturer's instructions. RNA-seq libraries were sequenced using the Illumina HiSeq 2500 system (Illumina). The raw reads for each library were deposited in DDBJ and are accessible through the Sequence Read Archive accession number DRA015177. Trimmed sequence reads that were prepared by Trimmomatic (51) were aligned to the *C. elegans* genome version WS274 (http://wormbase.org/) using HISAT2 under default parameters. Next, *featureCounts* was used for quantification, and edgeR was used for differential analysis.

### Gene ontology analysis

Upregulated genes were defined as satisfying a fold change of >2 and a false discovery rate of <0.05. Down-regulated genes were defined as satisfying a fold change of <0.5 and a false discovery rate of <0.05. The annotation of upregulated or down-regulated genes in loss of *emb-4* under each temperature condition was performed using WormCat (http://www.wormcat.com/) (52).

### CRISPR/Cas9

Knockout mutants were developed using a CRISPR protocol reported by the Mello Lab (53). The *scrm-4(chr65)* mutant contains a 461 bp deletion with a 13 bp insertion spanning from exon 1 to 2. Two crRNAs were used for targeting exon 1 (5′-CATGCAACCTGTGGAGATTT-[TGG]-3′) and exon 2 (5′-TCGTTGAATCCCCAACTCTG-[GGG]-3′), but the last three bases are not included in the crRNA because they are PAM sequences. We also used a short single stranded oligodeoxynuleotides (ssODN) donor (5′-AAAGTCGAGGTGCATGAAATCATGCAACCTGTGGATTAGCAGCTGAGAACGCTAGGTACTATTCGCCAGCGGTTTGGATTTCT-3′) that contains 35 bp of homology sequence at both ends cut by the two crRNAs and incorporates a 13 bp insertion sequence in the center. The injection solution containing Cas9 protein (Cat.#1081058, Integrated DNA Technologies (IDT), USA), tracrRNA (Cat. #1072532, IDT), two crRNAs (IDT), ssODN donor (IDT), and *rol-6(gf)* plasmid as a transgenic marker was microinjected into wild-type N2. After 24 F1 roller transgenic animals were single-picked and laid eggs, the genome of single worm was extracted and animals with deletion mutations were detected by following PCR. The F2 and subsequent generations also laid eggs after single picks and their genotypes were checked. The *scrm-4(chr65)* deletion mutation was identified by a sequence and a length of DNA fragments (WT: 1097 bp, deletion: 624 bp) amplified by PCR using primers KHR4106 (5′-ATACCAGAACAACCCCCGAC-3′) and KHR3525 (5′-CAAAAATGTTGCTCCCATCA-3′).

*asm-3(syb8325)* (SunyBiotech, China) was developed by using CRISPR-Cas9 method with wild-type N2 strain. *asm-3(syb8325)* mutant contains a 612 bp deletion spanning from exon 2 to 4 of a transcript W03G1.7a.1. The flanking sequences of the deletion site in the *asm-3(syb8325)* genome are 5′-TCCAGGAAAAAATTTTCCAG-3′ and 5′-GTAGGTTTTTTTTTTTTTTTTGACAA-3′, respectively.

### Oil red O staining

The oil red o staining protocol was performed basically as reported previously (20). Worms were synchronized and cultivated at a constant temperature; then, day-1 adult worms were harvested and washed three times in 1× PBS (pH 7.4) in a microcentrifuge tube. To permeabilize the worms, 120 µL of PBS and an equal volume of 2× MRWB buffer (160 mM KCl, 40 mM NaCl, 14 mM Na_2_EGTA, 1 mM spermidine-HCl, 0.4 mM spermine, 30 mM Na-PIPES, pH 7.4, and 0.2% beta-mercaptoethanol) containing 2% paraformaldehyde were added and gently shaken for 1 h. Then, the worms were washed with 1× PBS and incubated with 1.0 mL of 60% isopropanol for 15 min. A stock solution of 0.5% ORO dissolved in isopropanol was equilibrated over several days before being diluted to 60% with water, shaken for 1 h, and filtered through a 0.22 mm filter. This served as the “ORO staining solution”. From test tubes containing dehydrated *C. elegans*, 60% isopropanol was removed and 1 mL of “ORO staining solution” was added. The solution containing *C. elegans* was then rotated on a rotator for 30 min or overnight. The ORO solution was removed, and the worms were washed three times with 1.0 mL of 1× PBS containing 0.01% Triton X-100. Finally, the worms were mounted and bright-field images were acquired using a color digital camera (EOS kiss X9, Canon, Japan) mounted on a stereomicroscope (SZX12 Olympus, Japan).

### Image acquisition and quantification

All ORO staining image acquisitions were performed with constant camera settings and exposure times. For each worm, ORO fluorescence was acquired from color images using MetaMorph version 7.8 (Molecular Devices, USA), the intensity of the green channel was inverted, and the inverted value of unstained mock worms was subtracted. The average ORO fluorescence intensity per worm was estimated as the sum of the intensities within the enclosed region of the second and third intestinal cells.

### Statistical analysis

Cold tolerance and heat tolerance assays were performed on more than six plates and on at least three different days. The data values for each experiment are shown as dots in bar graphs. All error bars indicate SEM. Statistical analyses displayed in bar graphs were conducted using unpaired Welch's *t* tests to compare two groups, and one-way ANOVA followed by Dunnett's post hoc test or Tukey–Kramer test was performed for multiple comparisons. Single (*) and double (**) asterisks indicate *P* < 0.05 and *P* < 0.01, respectively. These tests were performed using Mac statistical analysis ver. 3 (Esumi, Japan). “Data S3 Raw_data.xlsx” file presents more details on raw data and statistical figures.

## Supplementary Material

pgae293_Supplementary_Data

## Data Availability

RNA-seq data have been deposited and released in the DDBJ BioProject with the accession number PRJDB14822 (https://ddbj.nig.ac.jp/resource/bioproject/PRJDB14822). RNA-seq data are deposited to the DDBJ Sequence Read Archive with the accession number DRA015177. The datasets generated during this study are available within this article and its supplementary information. Source data are provided with this article. Source data includes raw data and statistical analysis data.
