# Supplementary material for: The intron binding protein EMB-4 is an opposite regulator of cold and high temperature tolerance in Caenorhabditis elegans
Source: PNAS Nexus. 2024 Jul 26;3(8):pgae293. doi: 10.1093/pnasnexus/pgae293 (PMC11309393; doi:10.1093/pnasnexus/pgae293)
Supplement: pgae293_Supplementary_Data [file pgae293_supplementary_data.zip › PNASNEXUS-PNASNEXUS-2023-00546R-s03.docx]

**Supplementary Figure 1. Time course assay of heat and cold tolerance in wild-type and *emb-4.***

(A) The survival rate in the cold tolerance assay with wild-type N2 strain and *emb-4(hc60)*. Worms were cultivated at 20°C from egg to adult, and then they were transferred and incubated at 2°C for 6 h, 9h or 18 h, after which the survival rates were calculated. Number of assays ≥ 3. *n* = 406 and 317 worms for 2°C (6h); *n* = 551 and 199 worms for 2°C (9h); and *n* = 224 and 118 worms for 2°C (18h).

(B) The survival rate in the heat tolerance assay with wild-type N2 strain and *emb-4(hc60)*. Worms were cultivated at 20°C from egg to adult, and then they were transferred and incubated at 32°C for 9 h, 13h or 24 h, after which the survival rates were calculated. Number of assays ≥ 6. *n* = 713 and 305 worms for 32°C (9h); *n* = 480 and 219 worms for 32°C (13h); and *n* = 240 and 153 worms for 32°C (24h).

(C) *asm-3(tm2384)* and *scrm-4(tm624)* showed increased heat tolerance. The survival rate in the heat tolerance assay *asm-3(tm2384)* and *scrm-4(tm624)* mutants. Worms were cultivated at 15°C constantly from egg to adult, and then they were transferred and incubated at 31°C for 24 h, after which the survival rates were calculated. Number of assays ≥ 12. (*n* = 2656, 1887, and 1424 worms).

The error bars indicate SEMs. n.s. P ≥ 0.05; *P < 0.05; **P < 0.01. Comparisons were performed using Welch’s *t*-tests (A, B), or one-way ANOVA followed by Dunnett’s post hoc tests (C).
